# Mutations of *FAM111B* gene are not associated with Systemic Sclerosis

**DOI:** 10.1038/s41598-018-34341-7

**Published:** 2018-10-30

**Authors:** A. Gcelu, G. Deshpande, G. Shaboodien, T. F. Spracklen, A. Kalla, M. Tikly, B. M. Mayosi, B Hodkinson

**Affiliations:** 1 Division of Rheumatology, Department of Medicine, Groote Schuur Hospital and University of Cape Town, Cape Town, South Africa; 2 Cardiovascular Genetics Laboratory, Hatter Institute of Cardiovascular Research in Africa, Department of Medicine, Groote Schuur Hospital and University of Cape Town, Cape Town, South Africa; 3 Division of Rheumatology, Department of Medicine, Chris Hani Baragwanath Hospital, University of the Witwatersrand, Johannesburg, South Africa

## Abstract

Systemic sclerosis (SSc) is a prototypic systemic fibrotic disease with unclearly characterized genetic basis. We have discovered that mutations in family with sequence similarity 111, member B (*FAM111B*) gene cause hereditary fibrosing poikiloderma with tendon contractures, myopathy, and pulmonary fibrosis, a multisystem fibrotic condition with clinical similarities to SSc. This observation has established *FAM111B* as a candidate gene for SSc. Patients and Methods: Demographic and clinical characteristics of consenting adults with definite SSc were recorded. Blood DNA analysis was performed using the High-Resolution Melt technique, and samples with abnormal electropherograms were selected for Sanger sequencing to identify mutations. Ethnically-matched controls from the general South African population were used to verify the frequency of variants in *FAM111B*. Public databases such as 1000 Genomes and ExAC were also used to verify the frequency of variants in FAM111B. Results: Of 131 patients, 118 (90.1%) were female, and 78 (59.5%) were black Africans. Genetic analysis revealed two *FAM111B* genetic variants. The c.917 A > G variant (rs200497516) was found in one SSc patients, and one control, and was classified as a missense variant of unknown significance. The c.988 C > T variant (rs35732637) occurred in three SSc patients and 42/243 (17.3%) of healthy controls, and is a known polymorphism. Conclusion: One rare variant was found in a patient with SSc but has no functional or structural impact on the *FAM111B* gene. In this cohort, *FAM111B* gene mutations are not associated with SSc.

## Introduction

Systemic sclerosis (SSc) is a rare autoimmune disease characterized by widespread fibrosis, micro-vascular alterations and autoantibody production^1^. The disease is incurable, with a 5-year mortality of up to 50%, with respiratory failure accounting for over a third of deaths^2,3^. The pathogenesis is a poorly understood complex interaction between vascular dysfunction, dysregulation of the innate and adaptive immune systems, and excess activation of fibroblasts and related cells^4^.

Although SSc is not inherited in a Mendelian fashion, and heritability of the disease remains low, a family history of SSc is the strongest risk factor for developing the condition, and siblings of affected individuals have a 15-fold increased risk of SSc^5^. Frech *et al*. studied Utah Americans and demonstrated that the presence of SSc in first degree relatives increases susceptibility to SSc, Raynaud’s phenomenon, fibrotic lung disease and other autoimmune diseases^6^.

Recently, family with sequence similarity 111, member B (*FAM111B*) mutations were shown to cause a rare autosomal dominant disease, hereditary fibrosing poikiloderma^7^. This condition has clinical similarities to SSc, and is characterized by childhood-onset poikiloderma with tendon contractures, myopathy, and pulmonary fibrosis (POIKTMP). Whole exome sequencing revealed three single-nucleotide mutations on the *FAM111B* gene, presumably affecting the function of the gene and resulting in multisystem fibrosis.

We undertook this study on a cohort of South African SSc patients to determine the presence of *FAM111B* mutations.

## Patients and Methods

This cross-sectional study was conducted at rheumatology outpatient departments of two tertiary hospitals between June 2013 and December 2015. All patients met the 2013 American College of Rheumatology/European League of Arthritis and Rheumatism criteria for SSc and signed informed consent before participating^8^. Approval for the study was obtained from the University of Cape Town Human Research Ethics Committee and the University of the Witwatersrand Committee for Research on Human Subjects. All methods were performed in accordance with the relevant guidelines and regulations.

Demographic particulars and self-reported ethnic background, clinical details, the presence of serum autoantibodies (antinuclear factor (ANA), anti-topoisomerase 1 and anti-centromere antibodies), and investigations including chest x-ray (CXR), lung function tests, high resolution computed tomography (HRCT), barium studies, gastroscopy, and echocardiograms, were documented. Physical examination included the modified Rodnan skin score (mRSS). Eosophageal involvement was considered when a patient had a clinical complaint of dysphagia or heartburn; and/or barium swallow revealed esophageal dysmotility or reflux disease on gastroscopy. Pulmonary fibrosis was diagnosed when a patient presented with infiltrates or honeycombing on chest X ray and/or on high resolution computed tomography (HRCT) and had abnormal pulmonary function test (reduced forced vital and diffusion capacity). Pulmonary arterial hypertension (PAH) was defined as an elevated right ventricular systolic pressure (>45 mmHg) on echocardiography.

### Genetic analysis

Genomic DNA was extracted from peripheral leucocytes and mutational screening of *FAM111B* was performed using a High Resolution Melt (HRM) technique. An HRM reaction with a total volume of 25 ul/sample was prepared using 0.5 U GoTaq® Flexi DNA Polymerase (Promega, Madison, WI, USA), 1X Colorless GoTaq® Flexi Buffer (Promega), 3 mMMgCl2 (Promega), 0.8 μM dNTPs (Bioline, London, United Kingdom), 0.4x EvaGreen dye (Biotium, Hayward, CA, USA), 0.4, μM of each primer (forward and reverse) and 50 ng/ul DNA. HRM reactions were conducted using the RotorGene 6000 (Corbett Life Sciences – Qiagen, Venlo, Limber, Netherlands) and the cycling conditions were set at 95 °C for 10 minutes; 50 cycles of 95 °C for 5 seconds, 55 °C for 10 seconds and 72 °C for10 seconds; and a high resolution melt from 72 °C to 95 °C with 0.1 °C increases in temperature. Samples with abnormal electropherograms were selected for Sanger sequencing to identify mutations.

Samples were purified using Exonuclease I (New England Biolabs, Ipswich, MA, USA) and FastAPTM Thermosensitive Alkaline Phosphatase (Promega) using a Mastercycler® pro thermal cycler (Eppendorf, Hamburg, Germany); conditions were 37 °C for 1 hour and 75 °C for 15 minutes. Sequencing reactions were prepared using the BigDye® Direct Cycle Sequencing Kit (Applied Biosystems – Life Technologies, Carlsbad, CA, USA). Reaction conditions were 96 °C for 5 minutes and 25 cycles of 96 °C for 30 seconds, 50 °C for 15 seconds and 60 °C for 4 minutes. Sequencing products were analyzed using capillary electrophoresis with the ABI PRISM® 3130 × l Genetic Analyser (Applied Biosystems)^9^.

### Normal Controls

All variants that were identified in the SSc cohort were similarly screened in a cohort of 243 normal South African population controls made up of 107 (44.0%) Caucasians, 64 (26.3%) black Africans, and 72 (29.6%) individuals of mixed ancestry.

### Bioinformatic analysis

Public databases were also used to verify the frequency of variants in *FAM111B*. These included: 1000 Genomes (http://www.internationalgenome.org/) and ExAC (http://exac.broadinstitute.org/).

The missense variants were investigated using the following pathogenicity prediction tools: PolyPhen2 (http://genetics.bwh.harvard.edu/pph2/), SIFT http://sift.jcvi.org/), MutationTaster (http://www.mutationtaster.org/), MutationAssessor (http://mutationassessor.org/r3/), PROVEAN (http://provean.jcvi.org/index.php), AlignGVGD (http://agvgd.iarc.fr/), fathmm (http://fathmm.biocompute.org.uk/) and M-CAP (http://bejerano.stanford.edu/mcap/).

## Results

131 patients were genotyped, 13 men and 118 women, and the mean (SD) age of diagnosis was 47.2 (13.4) years (Table 1). The majority of patients were black Africans (59.5%). More than half (55.7%) had the diffuse cutaneous subtype (dcSSc). The vast majority had Raynaud’s phenomenon (90.1%), and were ANA positive (90.4%). As expected, skin scores in dcSSc patients were significantly higher compared to patients with the limited cutaneous subtype (lcSSc), and musculoskeletal manifestations (tendon friction rubs, joint contractures, muscle weakness and elevated creatinine kinase) were significantly more common in dcSSc than lcSSc patients. Over a quarter (26.7%) of patients had pulmonary fibrosis, and 12.2% had PAH.

**Table 1:** Demographic and clinical characteristics of 131 systemic sclerosis patients.

Characteristic	DcSSc (n = 73)	LcSSc (n = 58)	p-value [OR (95% CI)]
Ethnicity Black African - n (%)	47 (64.4)	31 (53.4)	ns
Mixed Ancestry - n (%)	21 (28.8)	23 (39.7)	ns
Caucasian - n (%)	4 (5.5)	3 (5.2)	ns
Indian - n (%)	1 (1.3)	1 (1.7)	ns
Female - n (%)	64 (87.7)	54 (93.1)	ns
Age at diagnosis -(years) mean (SD)	45.3 (62.1)	49.5(85.3)	0.06
Disease duration -(years) mean (SD)	6.8 (9.3)	7.5 (12.9)	ns
mRSS mean (SD)	16.7 (22.9)	10.9 (18.8)	0.002
Raynaud’s phenomenon - n (%)	66 (90.4)	52 (89.7)	ns
Digital Ulcers - n (%)	36 (49.3)	25 (43.1)	ns
Telengiectasia - n (%)	14 (19.2)	15 (25.9)	ns
Tendon Friction rub - n (%)	20 (27.4)	7 (12.1)	0.09 (2.78 (1.1–6.9)
Joint contractures - n (%)	35 (47.9)	8 (13.8)	< 0.001 (5.76 (2.4–13.2)
Muscle weakness - n (%)	22 (30.1)	9 (15.5)	0.02 [3.1 (1.3–7.9)
Elevated Creatinine Kinase - n (%)	25 (34.2)	11 (19.0)	0.004 [4.9 (2.1–11.6)
Pulmonary fibrosis - n (%)	22 (30.1)	13 (22.4)	ns
Pulmonary Artery Hypertension - n (%)	9 (12.3)	7 (12.1)	ns
Oesophageal disease - n (%)	45 (61.6)	44 (75.9)	ns
Other GIT* - n (%)	28 (38.4)	20 (34.5)	ns
Hypertension - n (%)	30 (41.1)	26 (44.9)	ns
Renal Crisis - n (%)	0 (0)	0 (0)	ns
Antinuclear Antibody positive - n (%)	58(79.5)	45(77.6)	ns
Anti-topoisomerase positive - n (%)	13/34 (38.2)	3/27 (11.1)	0.02 (5.0 (1.2–19.8))
Anti-centromere positive - n (%)	2/30 (0.1)	8/28 (28.6)	0.02 (0.17 (0.3–0.7)

DcSSc: diffuse cutaneous; Lcssc: limited cutaneous; ns: not significant; mRSS: modified Rodnan Skin Score; GIT: Gastro Intestinal Tract.

*Óther GIT involvement included malabsorption and chronic diarhhoea.

Genetic analysis of the SSc patients revealed two genetic variants of *FAM111B* in only four patients: c.917 A > G in one patient and c.988 C > T in three patients (Table 2). The c.917 A > G variant (rs200497516) was detected in one of the 243 healthy controls, and had a reported frequency of <0.01 in the 1000 Genomes and ExAC databases. This missense variant was thus classified as a genetic variant of unknown significance (GVUS). Although the amino acid was conserved across species, all bioinformatic pathogenicity prediction tools showed the variant to be benign and thus unlikely to have a structural or functional impact on the *FAM111B* (Table 3). The second variant, c.988 C > T variant (rs35732637) is a known polymorphism, having a reported frequency of >0.01 in the 1000 Genomes and ExAC databases, and was detected in 42/243 (17.3%) of the normal control population.

**Table 2:** *FAM111B* variants identified in SSc patients affected.

Chromosome position	Nucleotide change	Amino acid change	dbSNP identifier	SSc patient MAF	Control cohort MAF	1000 Genomes MAF	ExAC MAF
11:59125014	c.917 A > G	p.His306Arg	rs200497516	0.003	0.002	0.005	0.002
11:59125085	c.988 C > T	p.Leu330Leu	rs35732637	0.011	0.056	0.107	0.042

dbSNP: Single Nucleotide Polymorphism Database; MAF: Minor Allele Frequency.

**Table 3:** Protein function and structure prediction from population databases.

Variant/rs number	Mutation Taster prediction	PolyPhen2 HumDiv prediction	PolyPhen2 HumVar prediction	SIFT prediction	M-CAP prediction	fathmm	PROVEAN	Mutation Assessor	Align GVGD
c.917 A > G/rs200497516	Polymorphism (0.99)	Benign (0.006)	Benign (0.004)	Tolerated (0.15)	Not Scored	Tolerated (1.55)	Neutral (−2.31)	Low (1.04)	Class C25 (28.82)
c.988 C > T/rs35732637	NA	NA	NA	NA	Not scored	NA	NA	NA	NA

Abbreviations: NA, not applicable (because there is no change in amino acid).

## Discussion

To the best of our knowledge, this is the first study to investigate *FAM111B* mutations in SSc. Our findings of a missense variant and a known polymorphism with no functional or structural impact on *FAM111B* suggest that, in this cohort, *FAM111B* gene mutations are not associated with SSc. Thus, despite some clinical similarities, POIKTMP and SSc do not share genetic basis and are likely to have different pathogenetic pathways. The different pathogenetic pathways of these disorders are underscored by the absence of auto-antibodies in POIKTMP compared to SSc where there is definite evidence of auto-immunity.

The search for SSc susceptibility genes by candidate gene studies, and more recently by genome wide association studies (GWAS), have yielded numerous susceptibility genes, both in the major histocompatibility complex- human leucocyte antigen (MHC-HLA) region and outside this region^10^. Candidate genes for SSc fall into three groups that reflect the three main areas of disease expression and pathogenesis: immune responses, vasculopathy and fibrosis^11^. To date, the majority of gene mutations described are implicated in auto-immune dysregulation, with relatively few genetic variants pertaining to fibrosis or vasculopathy. It is possible that several polymorphisms occurring in an individual contribute to the risk, or that epigenetic alterations in gene expression underlie this complex disease.

The characteristics of patients in our cohort are comparable to those in the EULAR Scleroderma Trials and Research (EUSTAR) group database^12^, with some major differences. The predominance of the dcSSc subset in this cohort was also described in an earlier study of SSc in black South Africans where 65% of the patients had dcSSc compared to 37% of the EUSTAR cohort^13^. Less pulmonary fibrosis and PAH was diagnosed our cohort, probably because lung function tests, HRCT and echocardiograms were not routinely performed in all patients^12^. In addition, of the patients with antibody results, significantly fewer were anti-topoisomerase 1 and anti-centromere positive than described in the EUSTAR cohort. The absence of anti-centromere antibody was a feature of the other South African SSc study.

The majority of patients in our study were black Africans, and it is possible that mutations in *FAM111B* are present in other population groups, although this is unlikely based on the autoimmune basis of SSc compared to a hereditary origin of POIKTMP. Future studies should examine the role of the *FAM111B* mutation in the pathogenesis of related idiopathic conditions such as pulmonary fibrosis.

In conclusion, our study found one SSc patient with a missense variant of *FAM111B*, suggesting that, in this population, *FAM111B* is not associated with SSc.

## Data Availability

The datasets generated during and analysed during the current study are available from the corresponding author on reasonable request.
